# Improving Feature Representation Based on a Neural Network for Author Profiling in Social Media Texts

**DOI:** 10.1155/2016/1638936

**Published:** 2016-10-03

**Authors:** Helena Gómez-Adorno, Ilia Markov, Grigori Sidorov, Juan-Pablo Posadas-Durán, Miguel A. Sanchez-Perez, Liliana Chanona-Hernandez

**Affiliations:** ^1^Instituto Politécnico Nacional (IPN), Centro de Invetigación en Computación (CIC), Mexico City, Mexico; ^2^Instituto Politécnico Nacional (IPN), Escuela Superior de Ingeniería Mecánica y Eléctrica Unidad Zacatenco (ESIME-Zacatenco), Mexico City, Mexico

## Abstract

We introduce a lexical resource for preprocessing social media data. We show that a neural network-based feature representation is enhanced by using this resource. We conducted experiments on the PAN 2015 and PAN 2016 author profiling corpora and obtained better results when performing the data preprocessing using the developed lexical resource. The resource includes dictionaries of slang words, contractions, abbreviations, and emoticons commonly used in social media. Each of the dictionaries was built for the English, Spanish, Dutch, and Italian languages. The resource is freely available.

## 1. Introduction

Nowadays, the messages extracted from social media are being used for various purposes in the research area of natural language processing (NLP) and its practical applications. Tasks such as sentiment analysis, author profiling, author identification, opinion mining, plagiarism detection, and tasks related to computing text similarity, among others, rely on social media content in order to develop robust systems that help the decision making process in marketing, politics, education, forensics, and so forth.

Approaches based on neural networks (word embeddings) for unsupervised feature representation often do not perform data cleaning [1, 2], considering that the network itself would solve the related problems. These approaches treat special characters such as “,”, “.”, “!”, “?”, “#”, and username mentions (“@”) as a regular word [1, 3]. Still, in some works, where word embeddings are used, the use of basic data cleaning (stop words removal, URL filtering, removal of rare terms, etc.) significantly improves the feature representation and, consequently, the results of the classification task [4–6].

One of the problems with the content of social media messages is that usually they contain a large number and variety of nonstandard language expressions [7, 8]. The main problem is the nonstandardized writing style and irregularity of the language features used in this type of platforms: due to the short nature of the messages, most of the authors use a large vocabulary of slang words, abbreviations, and emoticons [9]. Slang words are not considered as a part of the standard vocabulary of a language, and they are mostly used in informal messages. Abbreviations are shortened forms of a word or name that are used in order to replace the full forms. Emoticons usually convey the current feeling of the message writer. The sets of slang words and abbreviations are specific for each language, and, therefore, the systems that perform processes over social media messages require specific dictionaries for each language. In general, emoticons are more universal.

The main goal of this work consists in development and evaluation of usefulness of a lexical resource, which contains dictionaries of abbreviations, contractions, slang words, and emoticons. This resource allows improvement of the feature representation obtained by a well-known neural network method: Doc2vec [1]. We consider that our dictionaries can help in achieving better results in tasks based on social media data, since they allow standardizing nonstandard language expressions that are used in a different way by different authors. We evaluate our hypothesis on the author profiling (AP) task, which aims at predicting the age and gender of an author of a given text [10]. In our research, we used Twitter messages. However, given that the writing style in social networks like Twitter, Facebook, and Instagram, among others, is similar, these dictionaries are useful for preprocessing and cleaning messages obtained from all these social networks.

The rest of the paper is structured as follows.  Section 2 describes related work.  Section 3 explains the methodology used for preparation of each dictionary and presents the structure of the dictionaries.  Section 4 describes a neural network-based feature representation.  Section 5 presents the case study for the author profiling task. Finally, Section 6 draws the conclusions from this work and points to the possible directions of future work.

## 2. Related Work

With the unprecedentedly growing amount of social media data available on the Internet and the rapid expansion in user-generated content, text preprocessing using corresponding lexical resources is becoming more and more crucial for subsequent accurate text analysis. In this section, we present several works that demonstrate the importance of the text preprocessing step and its usefulness in achieving better results for different NLP tasks, particularly for the tasks that use neural network-based feature representation.

The challenges that social media offers to NLP were discussed in detail in [11]. Further, we focus on the works (usually not related with word embeddings) that consider different preprocessing approaches in order to solve these challenges.

Clark and Araki [12] discuss the major problems related to processing social media messages written in English. The authors improved the performance of open source spellcheckers on Twitter data by developing a preprocessing system for automatically normalizing casual social media English. The authors report that when using their preprocessing system, average errors per sentence decreased from 15% to less than 5%.

The role of preprocessing has gained much importance, especially when dealing with social media data. It has been proven that an appropriate text preprocessing for the task of sentiment analysis (on social media data) significantly improves the results for this task [13–16]. Some of the most commonly used preprocessing steps include removing URLs, special characters, and repeated letters from a word; expanding abbreviations; removing stop words; handling negations; and performing stemming. Besides, preprocessing has proved to be useful even when dealing with other types of textual data, for example, source codes [17].

According to the overview papers of the author profiling (AP) task at PAN 2013 [18], PAN 2014 [19], and PAN 2015 [10], many participants carried out some kind of preprocessing, mainly for removing HTML tags from the tweets and secondly for handling hashtags, URLs, and username mentions in different ways.

In PAN 2015, there was only one team [20] that removed all character sequences representing emojis in the original tweets. Unlike emoticons, emojis are typically represented by Unicode characters, so, in this work, their use was replaced by unknown character markers. In the same work, the authors used a multi-input dependency parser [21] capable of identifying positive and negative emoticons for their further use as features in the classification process.

Several research teams that participated in the AP task at PAN (editions 2013, 2014, and 2015) exploited the use of emoticons and slang words as stylistic and content features, respectively. Some of these teams extracted emoticons using regular expressions [22, 23]. Other research teams built lexical resources for normalizing emoticons and Out Of Vocabulary (OOV) words with their corresponding normalized terms [24]; however, the authors did not publish these resources, making it difficult for others to reproduce their results. The rest of the teams that also used emoticons in their works did not mention any information concerning how the emoticons were identified in the corpus nor the source where they were obtained from (in case they used a dictionary) [25–29].

With respect to the use of slang words in the AP task, Goswami et al. [30] added slang words and the average length of sentences to the feature set, improving accuracy to 80.3% in age group identification and to 89.2% in gender detection. Farias et al. [28] and Diaz and Hidalgo [31] used dictionaries extracted from the web (http://www.chatslang.com/terms/common [last access: 01.07.2016]; all other URLs in this document were also verified on this date). However, these lists are very short in comparison with our emoticons and slang words dictionaries, which were collected manually from several web sources.

Regarding neural networks, several approaches have been proposed for vector-space distributed representations of words and phrases. These models are used mainly for predicting a word given a surrounding context. However, most of the authors indicate that distributed representations of words and phrases can also capture syntactic and semantic similarity or relatedness [1, 32, 33]. This particular behaviour makes these methods attractive to solve several NLP tasks; nevertheless, at the same time, it rises new issues, that is, dealing with unnormalized texts, which are typically present in social media forums such as Twitter, Facebook, and Instagram. Researchers have proposed several preprocessing steps in order to overcome this issue, which led to an overall performance increase. Yan et al. [4] enhanced system performance by approximately 2% using standard NLP preprocessing, which consists in tokenization, lowercasing, and removing stop words and rare terms. Rangarajan Sridhar [5] focused on the spelling issues in social media messages, which includes repeated letters, omitted vowels, use of phonetic spellings, substitution of letters with numbers (typically syllables), and use of shorthands and user created abbreviations for phrases. In a data-driven approach, Brigadir et al. [3] apply URL filtering combined with standard NLP preprocessing techniques.

As it can be seen, there are many works that tackle the problem of social media texts preprocessing; however, to the best of our knowledge, only few works based on a neural network for feature representation bothered to take into consideration the effect that data cleaning has on the quality of the representation (specially on social media data). In this work, we present our social media lexicon and demonstrate its usefulness for the author profiling task. This task aims at identifying the profile of the authors, that is, the age and gender, of social media messages. For our experiments, we used two corpora composed of Twitter messages obtained from the PAN competition in 2015 [10] and 2016 [34].

## 3. Creation of the Social Media Lexicon

We decided to develop the dictionaries for the four following languages, English, Spanish, Dutch, and Italian, because we needed to preprocess tweets written in these languages for the author profiling task at PAN 2015 [10]. The appropriate preprocessing of tweets was crucial for our system [35] performance, since our approach relied on correct extraction of syntactic *n*-grams [36]. We reviewed the tweets present in the PAN corpus and found an excessive use of shortened vocabulary, which can be divided into three main categories: slang words, abbreviations, and contractions.

The need to shorten the words emerged in order to save time and message length, and it became an essential part of an informal language. For example, in Twitter messages, one can find shortened expressions such as “xoxo” (kisses and hugs), “BFF” (best friend for ever), “LOL” (laughing out loud), “Argh!” (exclamation of disappointment), and “4ever” (forever). Moreover, we noticed that the same slang words (as well as other types of shortened expressions) are used differently by various authors. In order to standardize these shortened expressions, we grouped them in our dictionary of slang words. The dictionary of abbreviations is conformed by the set of shortened forms of words or phrases that can be used in both formal and informal languages. Our dictionary of abbreviations also includes acronyms, which are abbreviations that consist of the initial letters or parts of words or phrases. In order to give a few examples of abbreviations and acronyms included in our dictionaries, we can cite “St.” (street), “Ave.” (avenue), “Mr.” (mister), “NY” (New York), and “lb” (pound), among many others. The third category of the shortened vocabulary includes the contractions. A contraction is also a shortened version of a word, syllable, or phrase created by omitting internal letters. Different rules for different languages are established in order to create a contraction. Some examples of constructions for the English language are “let's” (let us), “aren't” (are not), “can't” (cannot), “who's” (who is), and so forth.

Moreover, we came across a large number of emoticons. Emoticon is a typographic display of a facial representation. The use of emoticons aims at making the text messages more expressive. We included in our emoticons dictionary two types of these graphic representations: western or horizontal (mainly from America and Europe) and eastern or vertical (mainly from east Asia). Western style emoticons are written from left to right, as if the head is rotated 90 degrees counterclockwise: “:-)” (smiley face), “:-/” (doubtful face), “:-o” (shocked face), among others. Eastern emoticons are not rotated, and the eyes often play a bigger role in the expression: “( $\hat{\;}\;v\;\hat{\;}$ )” (smiley face), “((+_+))” (doubtful face), and “(o.o)” (shocked face).

The compilation of the dictionaries is divided into three steps:

1. We searched on the Internet for freely available websites that were used as sources for the extraction of lists of slang words, contractions, and abbreviations.
2. From the selected sources, we manually extracted the lists of slang words, contractions, and abbreviations along with their corresponding meanings in each language (English, Spanish, Dutch, and Italian). We created different files with the same structure for each web source.
3. Once we had all the lists in different files, we proceeded to join all the files of the same nature. We formatted and cleaned each file; we also removed the duplicated entries. Finally, we manually checked the meanings of each entry in the dictionaries.

In order to evaluate the created social media lexicon, the initial idea consisted in using the Amazon Mechanical Turk [37] to collect crowdsourced judgments. However, unlike other NLP tasks such as word sense disambiguation and sentiment analysis, which can be effectively annotated by crowdsourced raters, the annotation of the created dictionaries requires specific knowledge of the Internet informal language. Therefore, as a reasonable alternative, the proposed resource was validated manually by 5 researchers who have linguistic backgrounds, speak multiple languages, and have an experience of working with social media content.

It is worth mentioning that all the dictionaries were extracted from the Internet sources with one exception—the dictionary of slang words for the Spanish language. This dictionary was expanded with the list of slang words obtained from [38]. In this work, the authors manually selected slang words from 21,000 tweets that had been previously downloaded using the hashtags of emotions (#feliz (happy), #felicidad (happiness), #alegría (joy), etc.) as search queries and the Tweepy (http://www.tweepy.org/) library for Python. Then, the researchers selected the words that are not present in the Spanish dictionary provided by the Freeling [39] tool. These words along with their contexts were manually revised by the authors in order to determine whether there exists an official word that conveys the corresponding meaning in order to include these entries into the slang words list. The authors provided us with the list of 200 slang words with their meanings, and it was added to the 739 slang words extracted from the web sources, making a total of 939 slang words for the Spanish language.

The links of the web pages that were used in order to extract the lists of slang words, contractions, and abbreviations for the English, Spanish, Dutch, and Italian languages, as well as the links of the pages used to obtain the list of emoticons for the English language, are presented in the Appendix. Since the meanings of the emoticons in English are the same as in Spanish, we translated them in order to create two dictionaries of emoticons (one for each language).

### 3.1. Description of the Dictionaries

Each dictionary is ordered alphabetically, and each file consists of two columns separated by a tabulation. The first column corresponds to a slang word, abbreviation, or contraction entry, according to the nature of the dictionary. The second column contains corresponding meanings of the entry. The meanings are separated by a fraction line and ordered from the most common to the least common ones.

The statistics for each dictionary is presented in Table 1, where we can see a high number of slang entities available for the English and Spanish languages; however, there are much less slang entities for Dutch and Italian. There are a large number of abbreviations for Dutch. The total number of entities in our social media lexicon is 7,259. The dictionaries are freely available on our website (http://www.cic.ipn.mx/~sidorov/lexicon.zip).

There are several ways of using the proposed resource. One option is to replace the occurrence of a shortened expression in the text by its corresponding meaning in the dictionary in order to standardize the use of the expression. In this case, the occurrence can be replaced by the most common meaning (the first meaning in the second column) or the meaning can be selected manually from the available options. Another way is to replace a shortened expression by a symbol in order to keep track of its occurrence and remove information related to the specific shortened expression. There is also an option of removing the nonstandard vocabulary instance identified by its presence in the dictionary.

## 4. Feature Representation Based on a Neural Network

**Figure 1:**
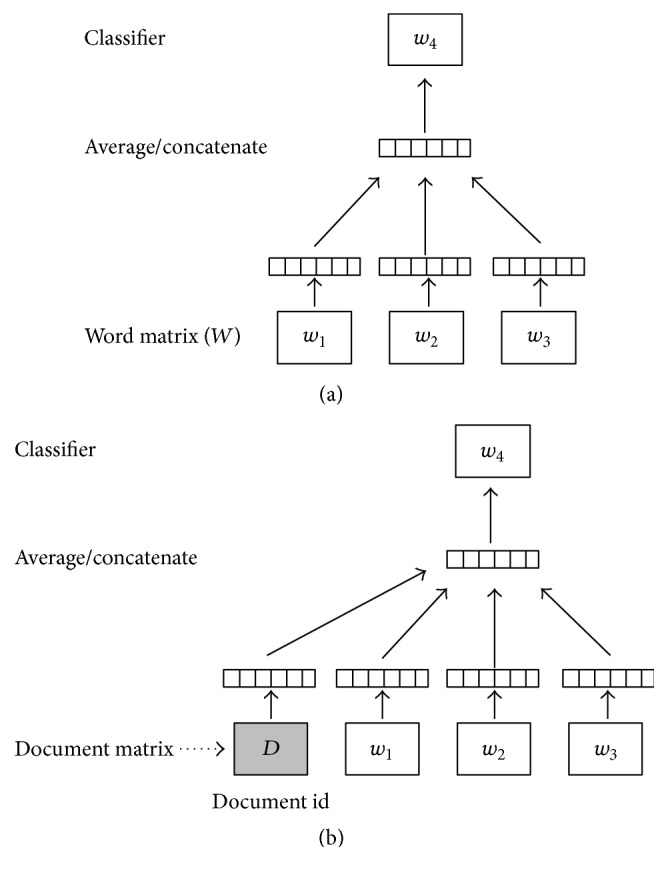
(a) Framework for learning word vectors and (b) framework for learning document vector.

In this work, we use a feature representation based on a neural network algorithm; that is, the features are learned in an automatic manner from the corpus. There are two ways to learn the feature representation of documents: (1) to generate documents vectors from word embeddings (or word vectors) or (2) to learn document vectors. In this work, we learn document vectors using the Doc2vec method following the previous research on learning word embeddings (Word2vec) [33].  Figure 1(a) shows the Word2vec method for learning word vectors. The Word2vec method uses an iterative algorithm to predict a word given its surrounding context. In this method, each word is mapped to a unique vector represented by a column in a matrix *W*. Formally, given a sequence of training words *w*_1_, *w*_2_, *w*_3_,…, *w*_*T*_, the training objective is to maximize the average of the log probability:

(1) $$\begin{array}{c} \frac{1}{T}\overset{T-k}{\underset{t=k}{\sum }}\log p\left(\;w_{t}\;|\;w_{t-k},\ldots ,w_{t+k}\;\right). \end{array}$$

For learning document vectors, the same method [1] is used as for learning word vectors (see Figure 1). Document vectors are asked to contribute to the prediction task of the next word given many contexts sampled from the document, in the same manner as the word vectors are asked to contribute to the prediction task about the next word in a sentence. In the document vector method (Doc2vec), each document is mapped to a unique vector represented by a column in document matrix. The word or document vectors are initialized randomly, but, in the end, they capture semantics as an indirect result of the prediction task. There are two models for distributed representation of documents: Distributed Memory (DM) and Distributed Bag-of-Words (DBOW).

The Doc2vec method implements a neural network-based unsupervised algorithm that learns feature representations of fixed length from texts (of variable length) [1]. In this work, we use a freely available implementation of the Doc2vec method included in the GENSIM (https://radimrehurek.com/gensim/) Python module. The implementation of the Doc2vec method requires the following three parameters: the number of features to be returned (length of the vector), the size of the window that captures the neighborhood, and the minimum frequency of words to be included into the model. The values of these parameters depend on the corpus. To the best of our knowledge, no work has been done on the author profiling task using the Doc2vec method. However, in previous work related to opinion classification task [40], a vector length of 300 features, a window size equal to 10, and a minimum frequency of 5 were reported. In order to narrow down the Doc2vec parameters search, we follow this previous research and conduct a grid search over the following fixed ranges: vector length [50,350], size of window [3,19], and minimum frequency [3,5]. Based on this grid search, we selected the Doc2vec parameters as shown in Table 2.

It is recommended to train the Doc2vec model several times with unlabeled data while exchanging the input order of the documents. Each iteration of the algorithm is called an epoch, and its purpose is to increase the quality of the output vectors. The selection of the input order of the documents is usually done by a random number generator. In order to ensure the reproducibility of the conducted experiments, in this work, we use a set of nine rules in order to perform the changes in the order the documents are input in each epoch (we run 9 epochs) of the training process. Considering the list of all unlabeled documents in the corpus *T* = [*d*_1_, *d*_2_,…, *d*_*i*_], we generate a new list of the documents with the different order *T*′ as follows:

(1) Use the inverted order of the elements in the set *T*, that is, *T*′ = [*d*_*i*_, *d*_*i*−1_,…, *d*_1_].
(2) Select first the documents with an odd index in ascending order and then the documents with an even index, that is, *T*′ = [*d*_1_, *d*_3_,…, *d*_2_, *d*_4_,…].
(3) Select first the documents with an even index in ascending order and then the documents with an odd index, that is, *T*′ = [*d*_2_, *d*_4_,…, *d*_1_, *d*_3_,…].
(4) For each document with an odd index, exchange it with the document of index *i* + 1, that is, *T*′ = [*d*_2_, *d*_1_, *d*_4_, *d*_3_,…].
(5) Shift in a circular way two elements to the left, that is, *T*′ = [*d*_*i*+2_, *d*_*i*+3_, *d*_*i*+4_,…, *d*_1_, *d*_2_,…].
(6) For each document with index *i*, exchange it with the document whose index is *i* + 3, that is, *T*′ = [*d*_4_, *d*_5_, *d*_6_, *d*_1_, *d*_2_, *d*_3_,…].
(7) For each document with index *i*, if *i* is a multiple of three, exchange it with the document next to it (*i* + 1), that is, *T*′ = [*d*_1_, *d*_2_, *d*_4_, *d*_3_, *d*_5_, *d*_7_, *d*_6_,…].
(8) For each document with index *i*, if *i* is a multiple of four, exchange it with the document next to it (*i* + 1), that is, *T*′ = [*d*_1_, *d*_2_, *d*_3_, *d*_6_, *d*_5_, *d*_4_,…].
(9) For each document with index *i*, if *i* is a multiple of three, exchange it with the document whose index is *i* + 2, that is, *T*′ = [*d*_1_, *d*_2_, *d*_5_, *d*_4_, *d*_3_, *d*_8_,…].

## 5. Case Study: The Author Profiling Task

The author profiling (AP) task consists in identifying certain aspects of a writer, such as age, gender, and personality traits, based on the analysis of text samples. The profile of an author can be used in many areas, for example, in forensics to obtain the description of a suspect by analyzing posted social media messages, or it can be used by companies to personalize the advertisements they promote in the social media or in the email interfaces [41].

In recent years, different methods have been proposed in order to tackle the AP task automatically, most of which use techniques from machine learning, data mining, and natural language processing. From a machine learning point of view, the AP task can be considered as a supervised multilabel classification problem, where a set of text samples *S* = {*S*_1_, *S*_2_,…, *S*_*i*_} is given, and each sample is assigned to multiple target labels (*l*_1_, *l*_2_,…, *l*_*k*_), where each position represents an aspect of the author (gender, age, personality traits, etc.). The task consists in building a classifier *F* that assigns multiple labels to unlabeled texts.

### 5.1. Experimental Settings

Since this work aims at evaluating the impact of our social media resource when using it in a preprocessing phase, we conducted the experiments with and without preprocessing the text samples, that is, replacing the shortened terms with their full representation. To perform the preprocessing, we made use of the dictionaries described earlier in Section 3.1 in the following manner:

1. Given a target shortened term or expression, we search for it in the corresponding dictionary.
2. If the shortened term is present in the dictionary, we replace it with the most common meaning (the first meaning in the second column). If the term is not present in the dictionary, we leave it unchanged.

We used a machine learning approach, which is divided into two stages: training and testing. In the training stage, a vector representation of each text sample is automatically obtained by a neural network framework, that is, *v* ^*i*^ = {*v*_1_, *v*_2_,…, *v*_*j*_}, where *v* ^*i*^ is the vector representation of the text sample *S*_*i*_. To obtain the vector representation of the text samples, a neural network-based distributed representation model is trained using the Doc2vec method [1]. The Doc2vec algorithm is fed with both labeled and unlabeled samples in order to learn the distributed representation of each text sample. We employed word *n*-grams with *n* ranging from 1 to 3 as input representation for the Doc2vec method. The algorithm takes into account the context of word *n*-grams, and it is able to capture the semantics of the input texts.

Then, a classifier is trained using the distributed vector representations of the labeled samples. We conducted the experiments using scikit-learn [42] implementation of the Support Vector Machines (SVM) and logistic regression (LR) classifiers, since these classifiers with default parameters have previously demonstrated a good performance on high-dimensional and sparse data. We generated different classification models for each one of the aspects of an author profile, that is, one model for the age profile and another for the gender profile.

In the testing phase, the vector representations of unlabeled texts are obtained using the distributed model built in the training stage; then the classifiers are asked to assign a label for each aspect of the author profile to each unlabeled sample.

We also performed an alternative evaluation of the proposed dictionaries using as baseline two state-of-the-art approaches for the AP task: character 3-grams and word unigrams (Bag-of-Words model) feature representations.

### 5.2. Datasets

In order to evaluate the proposed approach, we exploited two corpora designed for the 2015 and 2016 author profiling tasks at PAN (http://pan.webis.de/), which is held as part of the CLEF conference. The PAN 2015 corpus is composed of tweets in four different languages, English, Spanish, Dutch, and Italian, whereas the PAN 2016 corpus is composed of tweets in English, Spanish, and Dutch. Both corpora are divided into two parts: training and test. Each part consists of a set of labeled tweets that correspond to the age and gender. In addition, the PAN 2015 corpus includes several personality traits (extroverted, stable, agreeable, conscientious, and open). The full description of the corpora is given in Tables 3 and 4.

Both corpora are perfectly balanced in terms of gender groups. However, as can be seen comparing Tables 3 and 4, the number of instances in the PAN 2016 corpus is much higher than in the PAN 2015. Furthermore, the PAN 2016 corpus is more unbalanced in terms of age groups, and the number of these groups is higher than in the PAN 2015 corpus, which makes the PAN 2016 AP task more challenging.

The PAN 2015 and 2016 AP corpora are different in terms of the number of instances and the number and the distribution of age groups, which allows drawing more general conclusions when comparing the results of our approach with and without preprocessing.

Due to the policies of the organizers of the PAN AP task, only the 2015 and 2016 AP training datasets have been released. Therefore, in order to evaluate our proposal, we conducted the experiments on the training corpus under 10-fold cross-validation in two ways: (1) extracting the vectors from the whole dataset and (2) extracting the vectors from each fold of the cross-validation using only the training data. In both cases, we predicted the labels of unlabeled documents in each fold and calculated the accuracy of the predicted labels of all documents against the gold standard. It is worth mentioning that, at the PAN competition, the submitted systems are evaluated on the test data, which is only available on the evaluation platform (http://www.tira.io/) used at the competition. A preliminary version of the developed dictionaries was used in our submissions at PAN 2015 [35] and PAN 2016 [43].

### 5.3. Evaluation Results with Vectors Extracted from the Whole Dataset

Tables 5 [Table tab6] [Table tab7] [Table tab8] [Table tab9] [Table tab10]–11 present the age and gender classes accuracy obtained on the PAN 2015 and PAN 2016 corpora using two different classifiers (LR and SVM) with and without preprocessing. Here “LR-NP” is logistic regression without preprocessing; “LR-WP,” logistic regression with preprocessing; “SVM-NP,” SVM without preprocessing; “SVM-WP,” SVM with preprocessing; “D2V,” Doc2vec. The best results for each classifier (with/without preprocessing) are in bold. The best results for each feature set are underlined. Note that when conducting experiments on the PAN 2015 corpus for the age class, we only consider the Spanish and the English datasets, since for Dutch and Italian this class is currently not available. For the same reason, for the PAN 2016 corpus, we provide the results for the age and gender classes for the English and Spanish languages, while, for the Dutch language, the results are provided only for the gender class.

For the majority of cases, Doc2vec method outperforms the baseline approaches. However, for the Dutch and Italian datasets of PAN 2015, the character 3-grams approach provides higher accuracy. This can be explained by the fact that these corpora have a small number of instances, while the Doc2vec method requires a larger number of instances to build an appropriate feature representation.

We also observed that, for all the datasets, except for the Dutch 2016, the highest accuracy when using the Doc2vec method was obtained with higher-order *n*-grams as input representation (1 + 2-grams or 1 + 2 + 3-grams). This behaviour is due to the fact that these input representations allow the Doc2vec method to take into account syntactic and grammatical patterns of the authors with the same demographic characteristics.

Moreover, the obtained results indicate that, in most cases, system performance was enhanced when performing the preprocessing using the developed dictionaries, regardless of the classifier or the feature set. Furthermore, the highest results for each dataset for both the age and gender classes (except for the Spanish gender class on the PAN 2015 corpus) were obtained when performing the data preprocessing.

### 5.4. Evaluation Results with Vectors Extracted from Each Fold of the Cross-Validation Using Only the Training Data

In order to avoid possible overfitting produced by obtaining the vectors from the whole dataset, we conducted additional experiments extracting the vectors from each fold of the cross-validation using only the training data of the PAN 2015 and PAN 2016 corpora. This better matches the requirements of a realistic scenario, when the test data is not available at the training stage.

Tables 12 [Table tab13] [Table tab14] [Table tab15] [Table tab16]–17 present several results when the extraction of vectors from each fold in the cross-validation was done only with the training documents of this fold and not with the whole dataset. We follow the notations of the tables provided in the previous subsection.

The experimental results presented in Tables 12 [Table tab13] [Table tab14] [Table tab15] [Table tab16]–17 confirm the conclusion that higher classification accuracy is obtained when performing preprocessing using the developed dictionaries and that higher-order *n*-grams input representation for the Doc2vec method, allowing the inclusion of syntactic and grammatical information, in the majority of cases, outperforms the word unigrams input representation.

Furthermore, it can be observed that the results obtained when extracting the vectors from each fold of the cross-validation using only the training data are comparable to those when training on the whole dataset, ensuring no overfitting of the examined classifier. This also indicates that our method can be successfully applied under more realistic conditions, when there is no information on the test data at the training stage.

### 5.5. Statistical Significance of the Obtained Results

In order to ensure the contribution of our approach, we performed a pairwise significance test between the results of different experiments. We considered the Doc2vec approach with and without preprocessing, as well as the baseline approaches (character *n*-grams and Bag-of-Words). We used the Wilcoxon signed-ranks (Wsr) test [44] for computing the statistical significance of differences in results. The Wsr test is preferred over other statistical tests (such as Student's *t*-test) for comparing the output of two classifiers [45]. The Wsr is a nonparametric test that ranks the differences in performance of two classifiers in different datasets and compares the ranks for positive and negative differences. An important requirement of the Wsr test is that the compared classifiers are evaluated using exactly the same random samples, and at least five experiments are conducted for each method. In this work, we performed a stratified 10-fold cross-validation in each experiment, which allowed us to apply this test.

The significance levels are encoded as shown in Table 18. As is generally assumed, when *p* < 0.05, then we consider the systems to be significantly different from each other.  Table 19 presents the results of the Wsr test calculated for the English, Spanish, and Dutch languages. Here “D2V-NP” corresponds to the set of results obtained using the Doc2vec approach without preprocessing; “D2V-WP,” with preprocessing. The results obtained with the character 3-grams and Bag-of-Words approaches are represented as “Char. 3-grams-NP” and “Bag-of-Words-NP,” respectively.

From the significance test we can conclude that the Doc2vec approach with preprocessing obtained very significant (see Table 18) results as compared to both character *n*-grams and Bag-of-Words approaches without preprocessing for the considered languages. With respect to the Doc2vec method with and without preprocessing, the results are sometimes significant and sometimes are not. For example, in case of English, the “D2V-WP” obtained significant improvements over “D2V-NP”; however, in case of Spanish and Dutch, the results of “D2V-WP” are not significantly better than “D2V-NP.”

## 6. Conclusions and Future Work

Preprocessing of user-generated social media content is a challenging task due to nonstandardized and usually informal style of these messages. Furthermore, it is an essential step to a correct and accurate subsequent text analysis.

We developed a resource, namely, a social media lexicon for text preprocessing, which contains the dictionaries of slang words, contractions, abbreviations, and emoticons commonly used in social media. The resource is composed of the dictionaries for the English, Spanish, Dutch, and Italian languages. We described the methodology of the data collection, listed the web sources used for creating each dictionary, and explained the standardization process. We also provided information concerning the structure of the dictionaries and their length.

We conducted experiments on the PAN 2015 and PAN 2016 author profiling datasets. The author profiling task aims at identifying the age and gender of authors based on their use of language. We proposed the use of a neural network-based method for learning the feature representation automatically and a classification process based on machine learning algorithms, in particular, SVM and logistic regression. We performed a cross-validated evaluation of the PAN 2015 and PAN 2016 training corpora in two ways: (1) extracting the vectors from the whole dataset and (2) extracting the vectors from each fold of the cross-validation using only the training data. The experiments were conducted with and without preprocessing the corpora using our social media lexicon.

We showed that the use of our social media lexicon improves the quality of a neural network-based feature representation when used for the author profiling task. We obtained better results, in the majority of cases, for the age and gender classes for the examined languages when using the proposed resource, regardless of the classifier. We performed a statistical significance test, which showed that, in most cases, the results improvements obtained by using the developed dictionaries are statistically significant. We consider that these improvements were achieved due to the standardization of the shortened vocabulary, which is used in abundance in social media messages.

We noticed that there are some commonly used terms in social media that are not present in our web sources, specially for the English, Dutch, and Italian languages. Therefore, in future work, we intend to expand the dictionaries of slang words with manually collected entries for each language, as it was done for the Spanish slang words dictionary.

## Figures and Tables

**Table 1:** Number of entries in each dictionary.

Type of dictionary	Dutch	Italian	English	Spanish
Abbreviations	1,237	107	1,346	527
Contractions	15	56	131	11
Slang words	250	362	1,296	939
Emoticons	—	—	482	482

Total	1,520	525	3,255	1,959

**Table 2:** Parameters of the Doc2vec method for each language.

Parameter	Vector length	Window size	Minimum frequency
English	200	14	3
Spanish	350	10	3
Dutch	200	11	5
Italian	200	4	4

**Table 3:** Age and gender distribution over the PAN author profiling 2015 training corpus.

	English	Spanish	Dutch	Italian
*Age*				
18–24	58	22	—	—
25–34	60	46	—	—
35–49	22	22	—	—
50–xx	12	10	—	—

*Gender*				
Male	76	50	17	19
Female	76	50	17	19

*Total*	*152*	*100*	*34*	*38*

**Table 4:** Age and gender distribution over the PAN author profiling 2016 training corpus.

	English	Spanish	Dutch
*Age*			
18–24	26	16	—
25–34	135	64	—
35–49	181	126	—
50–64	78	38	—
65–xx	6	6	—

*Gender*			
Male	213	125	192
Female	213	125	192

*Total*	*426*	*250*	*384*

**Table 5:** Obtained results (accuracy, %) for age and gender classification on the PAN author profiling 2015 *English* training corpus under 10-fold cross-validation.

Feature set	Age			
	LR-NP	LR-WP	SVM-NP	SVM-WP
D2V (1-gram)	66.45	**66.45**	68.42	**69.73**
D2V (1 + 2-grams)	71.05	**74.34**	71.05	**72.36**
D2V (1 + 2 + 3-grams)	69.73	**70.39**	68.42	**70.39**
Character 3-grams	65.78	**67.76**	66.44	**67.10**
Bag-of-Words	**65.78**	**65.78**	**65.13**	**65.13**

Feature set	Gender			
	LR-NP	LR-WP	SVM-NP	SVM-WP

D2V (1-gram)	59.87	**66.44**	56.57	**69.07**
D2V (1 + 2-grams)	63.15	**69.73**	61.84	**71.05**
D2V (1 + 2 + 3-grams)	65.13	**69.07**	65.78	**71.71**
Character 3-grams	57.23	**61.84**	59.21	**62.50**
Bag-of-Words	**60.52**	56.57	**61.84**	55.26

**Table 6 tab6:** Obtained results (accuracy, %) for age and gender classification on the PAN author profiling 2015 *Spanish* training corpus under 10-fold cross-validation.

Feature set	Age			
	LR-NP	LR-WP	SVM-NP	SVM-WP
D2V (1-gram)	59.00	**62.00**	**62.00**	60.00
D2V (1 + 2-grams)	59.00	**65.00**	65.00	**69.00**
D2V (1 + 2 + 3-grams)	62.00	**66.00**	64.00	**66.00**
Character 3-grams	**66.00**	**66.00**	64.00	**67.00**
Bag-of-Words	**65.00**	62.00	**62.00**	60.00

Feature set	Gender			
	LR-NP	LR-WP	SVM-NP	SVM-WP

D2V (1-gram)	**65.00**	63.00	63.00	**66.00**
D2V (1 + 2-grams)	**68.00**	66.00	**66.00**	61.00
D2V (1 + 2 + 3-grams)	**71.00**	67.00	**71.00**	66.00
Character 3-grams	**73.00**	**73.00**	**75.00**	74.00
Bag-of- Words	**72.00**	71.00	**73.00**	72.00

**Table 7 tab7:** Obtained results (accuracy, %) for gender classification on the PAN author profiling 2015 *Dutch* training corpus under 10-fold cross-validation.

Feature set	Gender			
	LR-NP	LR-WP	SVM-NP	SVM-WP
D2V (1-gram)	61.76	**67.65**	61.76	**64.71**
D2V (1 + 2-grams)	64.71	**70.59**	67.65	**73.53**
D2V (1 + 2 + 3-grams)	**61.76**	58.82	**67.65**	64.71
Character 3-grams	**76.47**	**76.47**	**76.47**	**76.47**
Bag-of-Words	64.71	**67.65**	64.71	**70.59**

**Table 8 tab8:** Obtained results (accuracy, %) for gender classification on the PAN author profiling 2015 *Italian* training corpus under 10-fold cross-validation.

Feature set	Gender			
	LR-NP	LR-WP	SVM-NP	SVM-WP
D2V (1-gram)	**71.05**	**71.05**	**71.05**	68.42
D2V (1 + 2-grams)	**71.05**	**71.05**	68.42	**71.05**
D2V (1 + 2 + 3-grams)	78.95	**81.58**	78.95	**81.58**
Character 3-grams	**84.21**	**84.21**	**84.21**	**84.21**
Bag-of-Words	76.32	**78.95**	**78.95**	**78.95**

**Table 9 tab9:** Obtained results (accuracy, %) for age and gender classification on the PAN author profiling 2016 *English* training corpus under 10-fold cross-validation.

Feature set	Age			
	LR-NP	LR-WP	SVM-NP	SVM-WP
D2V (1-gram)	44.71	**44.84**	**41.78**	41.65
D2V (1 + 2-grams)	43.53	**44.37**	**42.82**	42.49
D2V (1 + 2 + 3-grams)	41.41	**46.71**	40.71	**44.13**
Character 3-grams	39.53	**41.78**	37.65	**42.96**
Bag-of-Words	**42.82**	39.44	**40.94**	39.91

Feature set	Gender			
	LR-NP	LR-WP	SVM-NP	SVM-WP

D2V (1-gram)	73.18	**75.59**	**72.71**	70.66
D2V (1 + 2-grams)	73.41	**78.64**	71.53	**74.88**
D2V (1 + 2 + 3-grams)	71.53	**77.46**	69.41	**76.76**
Character 3-grams	68.47	**73.24**	69.65	**71.83**
Bag-of-Words	69.18	**72.77**	67.76	**69.01**

**Table 10 tab10:** Obtained results (accuracy, %) for age and gender classification on the PAN author profiling 2016 *Spanish* training corpus under 10-fold cross-validation.

Feature set	Age			
	LR-NP	LR-WP	SVM-NP	SVM-WP
D2V (1-gram)	44.40	**46.00**	**44.80**	43.60
D2V (1 + 2-grams)	47.20	**52.40**	46.40	**51.20**
D2V (1 + 2 + 3-grams)	51.60	**56.00**	48.80	**57.20**
Character 3-grams	50.80	**52.00**	**48.00**	47.60
Bag-of-Words	**48.00**	47.60	44.00	**48.40**

Feature set	Gender			
	LR-NP	LR-WP	SVM-NP	SVM-WP

D2V (1-gram)	**71.20**	68.00	**67.60**	64.80
D2V (1 + 2-grams)	69.60	**71.60**	68.00	**70.40**
D2V (1 + 2 + 3-grams)	70.40	**75.60**	69.20	**73.60**
Character 3-grams	68.00	**69.60**	61.60	**63.20**
Bag-of-Words	**66.40**	63.60	58.80	**72.00**

**Table 11:** Obtained results (accuracy, %) for age and gender classification on the PAN author profiling 2016 *Dutch* training corpus under 10-fold cross-validation.

Feature set	Gender			
	LR-NP	LR-WP	SVM-NP	SVM-WP
D2V (1-gram)	74.74	**77.60**	71.09	**75.26**
D2V (1 + 2-grams)	70.83	**75.78**	71.09	**75.52**
D2V (1 + 2 + 3-grams)	73.44	**76.04**	70.31	**73.44**
Character 3-grams	**76.56**	72.66	**74.48**	72.92
Bag-of-Words	**74.48**	71.88	**74.74**	70.83

**Table 12:** Obtained results (accuracy, %) for age and gender classification using SVM classifier on the PAN author profiling 2015 *English* training corpus under 10-fold cross-validation with vectors extracted only from the training data.

Feature set	Age		Gender	
	SVM-NP	SVM-WP	SVM-NP	SVM-WP
D2V (1-gram)	66.57	**70.15**	59.38	**67.32**
D2V (1 + 2-grams)	**68.46**	65.55	63.13	**69.64**
D2V (1 + 2 + 3-grams)	66.97	**69.44**	66.96	**67.05**

**Table 13 tab13:** Obtained results (accuracy, %) for age and gender classification using SVM classifier on the PAN author profiling 2015 *Spanish* training corpus under 10-fold cross-validation with vectors extracted only from the training data.

Feature set	Age		Gender	
	SVM-NP	SVM-WP	SVM-NP	SVM-WP
D2V (1-gram)	**59.83**	56.67	67.00	**73.00**
D2V (1 + 2-grams)	68.33	**69.61**	67.00	**69.00**
D2V (1 + 2 + 3-grams)	69.50	**72.28**	65.00	**71.00**

**Table 14 tab14:** Obtained results (accuracy, %) for gender classification using SVM classifier on the PAN author profiling 2015 *Dutch* training corpus under 10-fold cross-validation with vectors extracted only from the training data.

Feature set	Gender	
	SVM-NP	SVM-WP
D2V (1-gram)	72.50	**75.00**
D2V (1 + 2-grams)	65.00	**70.00**
D2V (1 + 2 + 3-grams)	65.00	**72.50**

**Table 15 tab15:** Obtained results (accuracy, %) for age and gender classification using SVM classifier on the PAN author profiling 2016 *English* training corpus under 10-fold cross-validation with vectors extracted only from the training data.

Feature set	Age		Gender	
	SVM-NP	SVM-WP	SVM-NP	SVM-WP
D2V (1-gram)	41.89	**43.68**	**75.05**	73.27
D2V (1 + 2-grams)	43.06	**44.84**	76.75	**78.67**
D2V (1 + 2 + 3-grams)	42.83	**47.71**	75.77	**78.43**

**Table 16 tab16:** Obtained results (accuracy, %) for age and gender classification using SVM classifier on the PAN author profiling 2016 *Spanish* training corpus under 10-fold cross-validation with vectors extracted only from the training data.

Feature set	Age		Gender	
	SVM-NP	SVM-WP	SVM-NP	SVM-WP
D2V (1-gram)	**44.73**	42.94	73.17	**73.33**
D2V (1 + 2-grams)	**43.57**	43.21	74.42	**75.13**
D2V (1 + 2 + 3-grams)	44.53	**45.19**	74.74	**79.10**

**Table 17:** Obtained results (accuracy, %) for gender classification using SVM classifier on the PAN author profiling 2016 *Dutch* training corpus under 10-fold cross-validation with vectors extracted only from the training data.

Feature set	Gender	
	SVM-NP	SVM-WP
D2V (1-gram)	**74.18**	73.67
D2V (1 + 2-grams)	76.01	**76.78**
D2V (1 + 2 + 3-grams)	73.95	**75.97**

**Table 18:** Significance levels.

Symbol	Significance level	Significance
=	*p* > 0.05	Not significant
+	0.05 ≥ *p*> 0.01	Significant
++	0.01 ≥ *p* > 0.001	Very significant
+++	*p* ≤ 0.001	Highly significant

**Table 19:** Significance of results differences between pairs of experiments for the English, Spanish, and Dutch languages, where NP corresponds to “without preprocessing” and WP, “with preprocessing.”

Approaches	English		Spanish		Dutch	
	2015	2016	2015	2016	2015	2016
D2V-NP versus D2V-WP	+	+	=	=	=	=
Char. 3-grams-NP versus D2V-WP	+++	+++	+	+++	++	++
Bag-of-Words-NP versus D2V-WP	+++	+++	=	+++	+	+++

## Appendix

In this appendix, we provide the links of the web pages that were used to build our resource.

Sources links of each dictionary of the English language are the following.

*Abbreviations*

http://public.oed.com/how-to-use-the-oed/abbreviations/
http://www.englishleap.com/other-resources/abbreviations
http://www.macmillandictionary.com/us/thesaurus-category/american/written-abbreviations

*Contractions*

https://en.wikipedia.org/wiki/Wikipedia:List_of_English_contractions
http://grammar.about.com/od/words/a/Notes-On-Contractions.htm

*Slang Words*

http://www.noslang.com/
http://transl8it.com/largest-best-top-text-message-list/
http://www.webopedia.com/quick_ref/textmessageabbreviations.asp

Sources links of each dictionary of the Spanish language are the following.

*Abbreviations*

http://www.wikilengua.org/index.php/Lista_de_abreviaturas_A
http://www.reglasdeortografia.com/abreviaturas.htm
http://buscon.rae.es/dpd/apendices/apendice2.html

*Contractions*

http://www.gramaticas.net/2011/09/ejemplos-de-contraccion.html

*Slang Words*

http://spanish.about.com/od/writtenspanish/a/sms.htm
https://www.duolingo.com/comment/8212904
http://www.hispafenelon.net/ESPACIOTRABAJO/VOCABULARIO/SMS.html

Sources links of each dictionary of the Dutch language are the following.

*Abbreviations*

https://nl.wikipedia.org/wiki/Lijst_van_afkortingen_in_het_Nederlands

*Contractions*

https://en.wiktionary.org/wiki/Category:Dutch_contractions
https://www.duolingo.com/comment/5599651

*Slang Words*

http://www.welingelichtekringen.nl/tech/398386/je-tiener-op-het-web-en-de-afkortingen.html
http://www.phrasebase.com/archive2/dutch/dutch-internet-slang.html
https://nl.wikipedia.org/wiki/Chattaal

Sources links of each dictionary of the Italian language are the following.

*Abbreviations*

http://homes.chass.utoronto.ca/~ngargano/corsi/corrisp/abbreviazioni.html
http://www.paginainizio.com/service/abbreviazioni.htm

*Contractions*

http://www.liquisearch.com/contraction_grammar/italian
https://en.wikipedia.org/wiki/Contraction_/%28grammar/%29#Italian

*Slang Words*

https://it.wikipedia.org/wiki/Gergo_di_Internet#Il_gergo_comune_di_internet
http://unluogocomune.altervista.org/elenco-abbreviazioni-in-uso-nelle-chat/
http://www.wired.com/2013/11/web-semantics-gergo-di-internet/

Sources links of the dictionary of emoticons are the following.

https://en.wikipedia.org/wiki/List_of_emoticons
http://pc.net/emoticons/
http://www.netlingo.com/smileys.php
